# Factors affecting male-to-female ratio at birth in frozen-thawed embryo transfer cycles: a large retrospective cohort study

**DOI:** 10.3389/fendo.2023.1188433

**Published:** 2023-09-20

**Authors:** Tong Du, Qin Xie, Jing Ye, Xindi Wang, Jiaxin Qiu, Zheng Yan, Suqun Zhang, Dong Zhao, Jiaying Lin, Bin Li

**Affiliations:** ^1^ Department of Assisted Reproduction, Shanghai Ninth People’s Hospital, Shanghai Jiao Tong University School of Medicine, Shanghai, China; ^2^ Department of Integrative Physiology and Biochemistry, University of Colorado at Boulder, Boulder, NV, United States; ^3^ Department of Obstetrics and Gynaecology, Shanghai Ninth People’s Hospital, Shanghai Jiao Tong University School of Medicine, Shanghai, China

**Keywords:** blastocyst transfer, FET, ICSI, IVF, male-to-female ratio

## Abstract

**Background:**

ICSI (intracytoplasmic sperm injection) leads to a reduced male-to-female ratio at birth, whereas blastocyst transfer results in an increased male-to-female ratio. However, limited knowledge exists regarding the impact of these factors on the live birth rate for each gender. This study aimed to investigate the influence of patient characteristics and treatment parameters on the live birth rate for each gender, as well as the ultimate male-to-female ratio at birth in frozen-thawed embryo transfer (FET) cycles.

**Method:**

This retrospective cohort study involved a total of 28,376 FET cycles and 9,217 subsequent deliveries, spanning from January 2003 to December 2015. The study consisted of two parts. First, logistic regression models were constructed to determine the factors influencing the male-to-female ratio among babies born after FET. Second, we aimed to investigate the mechanisms underlying this sex ratio imbalance by analyzing data from all transfer cycles. Generalized estimated equations were employed to assess the impact of risk factors on rates of male and female live births separately.

**Results:**

ICSI resulted in a lower proportion of male offspring compared to *in vitro* fertilization (IVF) (50.1% vs. 53.7%, aOR: 0.87, 95% CI: 0.80-0.96). Conversely, blastocyst transfer yielded a higher proportion of male offspring than cleavage-stage embryo transfer (58.7% vs. 51.6%, aOR: 1.32, 95% CI: 1.17-1.48). Analysis of all cycles indicated that ICSI resulted in a reduced likelihood of male live birth in comparison to IVF (19.8% vs. 21.6%, aOR: 0.90, 95% CI: 0.83-0.97). However, the transfer of blastocysts rather than cleavage-stage embryos not only increased the chance of male live birth (26.9% vs. 20.2%, aOR: 1.70, 95% CI:1.56-1.85) but also facilitated female live birth (20.3% vs. 19.3%, aOR: 1.26, 95% CI: 1.15-1.39).

**Conclusion:**

ICSI was associated with a reduction in the male-to-female sex ratio and a lower rate of male live births, while blastocyst transfer was associated with an increased male-to-female sex ratio at birth and a higher rate of male live births.

## Introduction

Ever since the first birth accomplished through *in vitro* fertilization (IVF) in 1978 (1), the advent of IVF has brought approximately 10 million infants into the world (2). In recent years, IVF births have accounted for approximately 1% and 2% of all deliveries in China and in the United States, respectively (3, 4). Nevertheless, anxiety lingers in society regarding the potential repercussions of this artificial mode of conception, particularly with regard to its impact on the resulting offspring.

The general male-to-female ratio of all births (i.e., the proportion of male newborns) tends to hover at approximately 51.2% (male:female=105:100) (5); this ratio plays a critical role in facilitating social equilibrium and warding off undesirable socioeconomic consequences (6). The ratio itself, in turn, is dependent on a multitude of factors spanning the realms of biology (e.g., the age and body mass index (BMI) of the mother and father), the environment (e.g., exposure to pollutants and pesticides), society (e.g., gender selection and selective abortion), and economics (e.g., economic downturns and stressors) (7–17).

Additionally, the impact of procedures used in assisted reproduction technology (ART) on the male-to-female ratio cannot be disregarded. As presented in **Table 1**, studies have suggested that intracytoplasmic sperm injection (ICSI) may increase the proportion of female offspring by 2.2–5.4% compared to IVF (19–21, 24). In contrast, blastocyst transfer has been found to be associated with a sex-ratio imbalance, resulting in 2.7–3.8% more male offspring (19, 21, 25–27). Although these two outcomes have been extensively documented, there has been limited research investigating the relationships of various factors involved in ART (such as the underlying characteristics of infertile couples, reproductive history, and treatment interventions) with male-to-female ratio at birth. Furthermore, it remains unknown whether these factors have a gender-specific impact on the live birth rate.

**Table 1:** Summary of previous and current studies on sex ratio^†^.

Study	Region	Study design	Period	Sample size	Reported sex ratio ^†^				
					Overall	Cleavage transfer	Blastocyst transfer	IVF	ICSI
Cirkel et al., 2018 (18)	Germany	Population-based	1997–2009	59,628	50.8%^*^	NA	NA	52.2%	50.0%
Dean et al., 2010 (19)	Australia and New Zealand	Population-based	2002–2006	13,368	51.3%	49.9%	54.1%	53.0%	50.0%
Bu et al., 2014 (20)	China	Cohort	2002–2012	121,247	51.8%	51.4%	54.9%	52.3%	49.7%
Luke et al., 2009 (21)	United States	Population-based	2005	15,164	52.5%	48.9%	51.6%	51.4%	48.8%
Arikawa et al., 2016 (22)	Japan	Cohort	2007–2012	27,158	50.9%	49.9%	52.9%	53.1%	47.7–48.2%
Ishihara et al., 2014 (23)	Japan	Population-based	2008–2010	47,895	52.6%^*^	50.0%, 50.2%^*#^	53.1%, 53.9%^*#^	NA	NA
Current study	China	Cohort	2003–2015	10,576	52.3% (6982/6380)	51.2% (5864/5580)	58.3% (1118/800)	53.1% (5026/4436)	50.2% (1956/1944)

^†^Sex ratio at birth is defined as the proportion of male offspring.

^*^ Values calculated in the form of original male-to-female odds.

^#^For fresh and frozen transfer cycles, respectively.

IVF, in vitro fertilization; ICSI, intracytoplasmic sperm injection; NA, not available.

The objective of this study was to analyze the potentially differential impact of various risk factors on live birth rate for each gender in couples undergoing frozen-thawed embryo transfer (FET).

## Methods

### Ethical approval

This study was approved by the Institutional Review Board of Shanghai Ninth People’s Hospital, Shanghai Jiao Tong University School of Medicine (SH9H-2021-T271-1).

### Study design and population

This retrospective cohort study was conducted at the Department of Assisted Reproduction of Shanghai Ninth People’s Hospital, Shanghai Jiao Tong University School of Medicine. The study screened patients who underwent FET treatment between January 2003 and December 2015, ensuring that complete information was available. In the analyses of data on live births, patients who delivered twins of different genders and those with multiple deliveries during the study period were excluded.

### Treatment

The IVF/ICSI procedures followed have been described in our previous publications (28). In brief, fertilization was performed either by IVF or by ICSI 4–6 hours after oocyte retrieval. On Day 3, embryos were evaluated according to the Cummins criteria (29). Embryos graded as I and II were cryopreserved on Day 3, while culture of embryos classified as III/IV was extended until Day 7 to enable the selection of morphologically good blastocysts using the Gardner and Schoolcraft grading system; blastocysts meeting the minimum requirement of 3CC were considered eligible for cryopreservation on Day 7 (29, 30).

Endometrial preparation and the FET procedure have previously been described in detail (28). In brief, endometrial preparation was conducted using modified natural cycles, mild stimulation cycles, or hormonal therapy treatments for patients with regular menstrual cycles, irregular menstrual cycles, or a history of thin endometrium, respectively. A progestin supplement was administered until 10 weeks of gestation after achievement of pregnancy.

### Statistical analysis

Data are presented in the form of % (n/N). Between-groups differences were assessed using the Chi-square test or Fisher’s exact test, whichever was appropriate. Statistical significance was determined by a *P*-value <0.05, and odds ratio (ORs) and 95% confidence intervals (CIs) were calculated as indicators of statistically significant effects. Data analysis was conducted using the SPSS software package, version 23.0 (SPSS Inc., Chicago, USA).

Factors analyzed in this study included maternal age (≤29, 30-34, 35-37, 38-40, 41-43, or ≥44), maternal BMI (<18.5, 18.5-24, or >24), partner age (≤29, 30-39, 40-49, or ≥50), duration of infertility (≤1, 2-4, 5-9, ≥10 years), previous miscarriages (0, 1, or ≥2), previous ectopic pregnancy (yes or no), tubal factor infertility (yes or no), PCOS (yes or no), endometriosis (yes or no), male factor infertility (yes or no), treatment year (2003-2009, 2010-2011, 2012-2013, or 2014-2015), fertilized method (IVF or ICSI), endometrial preparation for FET (natural cycle, hormone therapy treatment, or mild stimulation), endometrial thickness at transfer (≤8, 8-15, or ≥15 mm), number of embryos transferred (1, 2, or 3), and embryonic stage at transfer (cleavage or blastocyst).

First, we analyzed the male-to-female ratio among live-born babies. Univariate logistic regression was employed to examine the potential effects of various characteristics on the male-to-female ratio. Multivariate logistic regression analysis, using the conditional backward method, was conducted to identify variables with a significant influence on the male-to-female ratio among offspring, and to calculate adjusted odds ratios (aORs) and 95% confidence intervals (CIs). The significance of the models was assessed based on the −2 log likelihood, and their goodness of fit of models was evaluated using Nagelkerke’s R^2^.

Second, we investigated the impact of the aforementioned risk factors on live birth rate for each gender across all transfer cycles. Generalized estimated equation (GEE) models were conducted to address the issue of clustered data (multiple cycles for the same woman), and to calculate ORs and 95% CIs. Significant variables (defined as *P* < 0.2 in a Chi-square test or Fisher’s exact test) were included in multivariate models. GEE models were evaluated based on the quasi-likelihood under independence model criterion.

Finally, we investigated the relationships between each of the aforementioned characteristics and the gender of newborns in all cases of live births involving twins. A multivariate logistic regression model (using the simultaneous entry method) was constructed to calculate aORs and 95% CIs.

## Results

**Figure 1:**
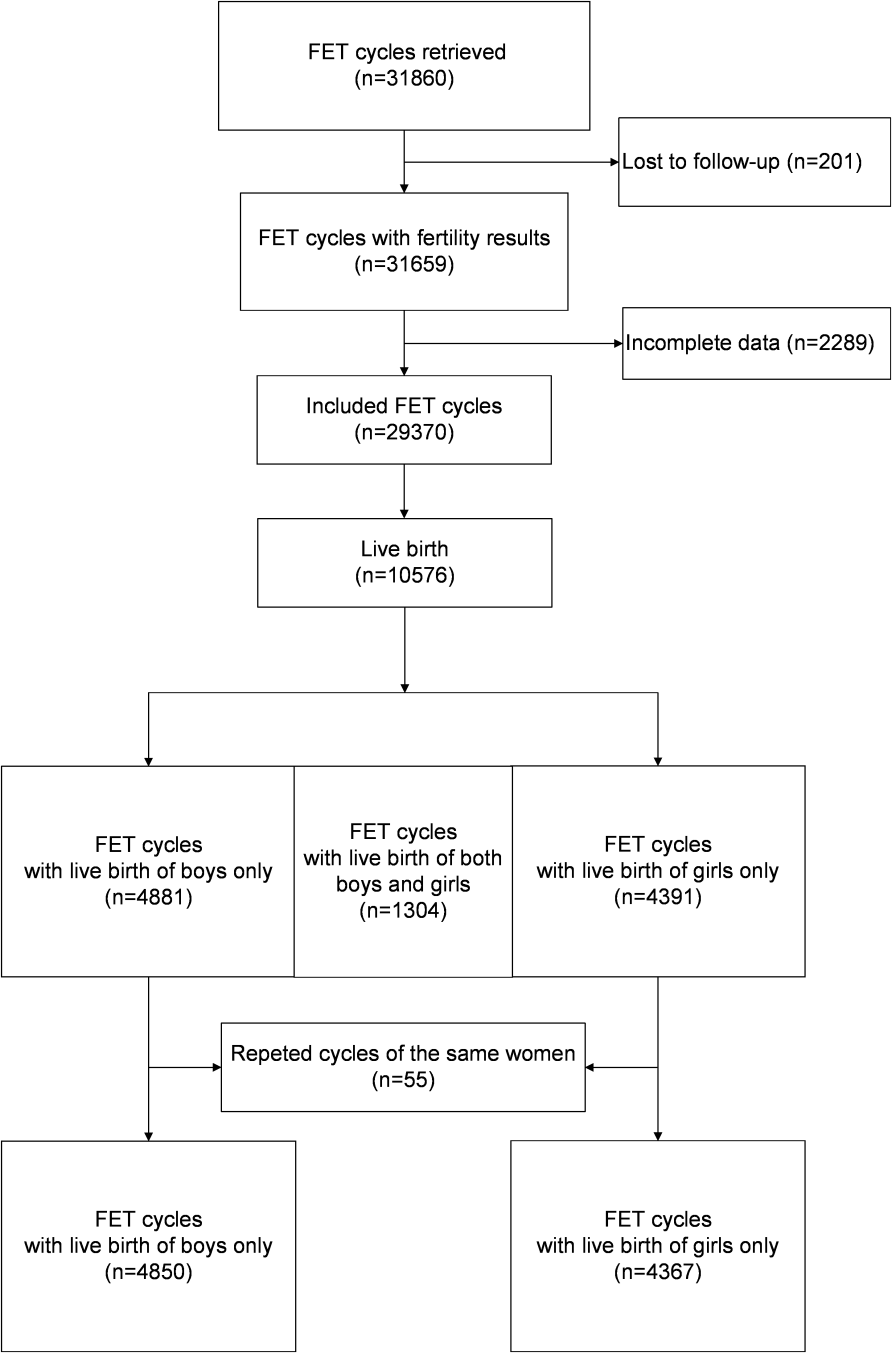
Flow chart of the study.

**Figure 1** illustrates the analysis of 29,370 cycles conducted between January 2003 and December 2015, with the aim of examining the relationships between various risk factors and the live birth rate for each gender. Among these cycles, a total of 10,576 cycles resulted in live births. After the exclusion of twin deliveries involving babies of different genders (*n* = 1,304) and repeated deliveries by the same women (*n* = 55), a total of 9,217 deliveries were included for assessment of the associations between the risk factors and the male-to-female ratio at birth; these consisted of 5,639 male babies and 5,047 female babies.

### Association between risk factors and male-to-female ratio among all live-born babies

**Table 2** presents the male-to-female ratio among all live-born babies when stratified based on patient and treatment characteristics. In comparison to IVF, ICSI resulted in a lower proportion of male offspring (50.1% vs. 53.7%, aOR: 0.87, 95% CI: 0.80–0.96). However, blastocyst transfer was associated with a higher likelihood of male offspring compared to cleavage embryo transfer (58.7% vs. 51.6%, aOR: 1.32, 95% CI: 1.17–1.48).

**Table 2:** Sex ratio by patient characteristics and treatment parameters for all live births.

	Male offspring, n (%)	Live births, n (N=9217)	*P*	Crude OR (95% CI)	Adjusted OR (95% CI)
Maternal age			0.886		
≤29	1478 (52.2)	2834		Reference	
30-34	2223 (52.7)	4219		1.02 (0.93-1.12)	
35-37	748 (53.6)	1396		1.06 (0.93-1.20)	
38-40	306 (53.0)	577		1.04 (0.87-1.24)	
41-43	85 (49.1)	173		0.89 (0.65-1.21)	
≥44	10 (55.6)	18		1.15 (0.45-2.91)	
Maternal BMI			0.56		
<18.5	3405 (52.5)	6491		0.98 (0.86-1.11)	
18.5-24	575 (51.9)	1108		Reference	
>24	870 (53.8)	1618		1.05 (0.95-1.18)	
Male partner age			0.515		
≤29	917 (51.4)	1784		Reference	
30-39	3300 (52.9)	6233		1.06 (0.96-1.18)	
40-49	593 (53.1)	1116		1.07 (0.92-1.25)	
≥50	40 (47.6)	84		0.86 (0.56-1.33)	
Duration of infertility (years)			0.854		
≤1	894 (53.2)	1618		Reference	
2-4	2437 (52.3)	4656		0.97 (0.87-1.08)	
5-9	1268 (53.0)	2392		0.99 (0.88-1.13)	
≥10	251 (51.4)	488		0.93 (0.76-1.14)	
Previous miscarriages			0.566		
0	2622 (52.1)	5029		Reference	
1	1303 (53.0)	2459		1.04 (0.94-1.14)	
≥2	925 (53.5)	1729		1.06 (0.95-1.18)	
Previous ectopic pregnancy			0.781		
No	4383 (52.7)	8322		Reference	
Yes	467 (52.2)	895		0.98 (0.85-1.13)	
Tube factor			0.394		
No	2418 (52.2)	4634		Reference	
Yes	2432 (53.1)	4583		1.04 (0.96-1.12)	
PCOS			0.083		
No	4541 (52.9)	8590		Reference	
Yes	309 (49.3)	627		0.87 (0.74-1.02)	
Endometriosis			0.550		
No	4473 (52.5)	8515		Reference	
Yes	377 (53.7)	702		1.05 (0.90-1.22)	
Male factor			0.009		
No	3272 (53.6)	6105		Reference	
Yes	1578 (50.7)	3112		**0.89 (0.82-0.97)**	
Treatment year			0.003	0.97 (0.95-0.99)	
2003-2009	283 (52.1)	543			
2010-2011	984 (56.6)	1737			
2012-2013	2217 (51.5)	4303			
2014-2015	1366 (51.9)	2634			
Fertilization method			0.001		
IVF	3477 (53.7)	6474		**Reference**	**Reference**
ICSI	1373 (50.1)	2743		**0.86 (0.79-0.95)**	**0.87 (0.80-0.96)**
Endometrial preparation for FET			0.086		
Natural cycle	1418 (53.7)	2643		Reference	
Mild stimulation	1615 (53.4)	3023		0.99 (0.89-1.10)	
HRT	1817 (51.2)	3551		0.91 (0.82-1.00)	
Endometrial thickness at transfer, mm			0.238		
≤8	316 (54.1)	584		Reference	
8-15	4146 (52.3)	7931		1.08 (0.91-1.27)	
≥15	388 (55.3)	702		1.13 (0.97-1.32)	
Embryo stage at transfer			<0.001		
Cleavage stage	4057 (51.6)	7865		**Reference**	**Reference**
Blastocyst stage	793 (58.7)	1352		**1.33 (1.19-1.50)**	**1.32 (1.17-1.48)**
No. of embryos transferred			0.516		
1	554 (54.3)	1020		Reference	
2	4145 (52.4)	7908		0.93 (0.81-1.06)	
3	151 (52.2)	289		0.92 (0.71-1.20)	

Values are presented in the form number (percentage). BMI, body mass index; PCOS, polycystic ovary syndrome; OPU, ovum pick-up; IVF, in vitro fertilization; ICSI, intracytoplasmic sperm injection; FET, frozen-thawed embryo transfer; HRT, hormone replacement therapy; COR, crude odds ratio; AOR, adjusted odds ratio; CI, confidence interval.

Bold indicates statistical significance.

### Association between risk factors and newborn gender among all transfer cycles

**Table 3** presents a comprehensive overview of the male and female live birth rates based on patient and treatment characteristics. **Table 4** shows the results of multivariate analysis. In the adjusted analysis, it was observed that women undergoing ICSI had a reduced likelihood of male live birth (aOR: 0.90, 95% CI: 0.83–0.97). The chances of both male and female live birth were increased when blastocysts were transferred rather than cleavage-stage embryos (male: aOR 1.70, 95% CI 1.56–1.85; female: aOR 1.26, 95% CI 1.15–1.39).

**Table 3:** Rates of births of male and female offspring by patient characteristics and treatment parameters for all transferred cycles.

	Cycles, n	Male offspring, n (%)	*P*	Female offspring, n (%)	*P*
Total	29370	6185 (21.1)		5695 (19.4)	
Maternal age			<0.001		<0.001
≤29	7498	1978 (26.4)		1855 (24.7)	
30-34	12215	2851 (23.3)		2624 (21.5)	
35-37	4654	912 (19.6)		807 (17.3)	
38-40	2615	338 (12.9)		301 (11.5)	
41-43	1552	95 (6.1)		99 (6.4)	
≥44	836	11 (1.3)		9 (1.1)	
Maternal BMI			0.155		0.005
<18.5	20662	4355 (21.1)		682 (20.8)	
18.5-24	3276	724 (22.1)		4031 (19.5)	
>24	5432	1106 (20.4)		982 (18.1)	
Male partner age			<0.001		<0.001
≤29	4725	1219 (25.8)		1166 (24.7)	
30-39	18818	4220 (22.4)		3848 (20.4)	
40-49	5258	700 (13.3)		632 (12.0)	
≥50	569	46 (8.1)		49 (8.6)	
Duration of infertility (years)			<0.001		<0.001
≤1	4886	1183 (24.2)		1070 (21.9)	
2-4	14320	3184 (22.0)		2933 (20.5)	
5-9	7806	1552 (19.9)		1405 (18.0)	
≥10	2358	302 (12.8)		287 (12.2)	
Previous miscarriages			<0.001		<0.001
0	15166	3366 (22.2)		3154 (20.8)	
1	7818	1659 (21.2)		1503 (19.2)	
≥2	6386	1160 (18.2)		1038 (16.3)	
Previous ectopic pregnancy			0.003		0.002
No	26781	5580 (20.8)		5132 (19.2)	
Yes	2589	605 (23.4)		563 (21.7)	
Tubal factor			0.751		0.406
No	14759	3097 (21.0)		2890 (19.6)	
Yes	14611	3088 (21.1)		2805 (19.2)	
PCOS			<0.001		<0.001
No	27704	5764 (20.8)		5264 (19.0)	
Yes	1666	421 (25.3)		431 (25.9)	
Endometriosis			0.222		0.834
No	27336	5735 (21.0)		5297 (19.4)	
Yes	2034	450 (22.1)		398 (19.6)	
Male factor			0.994		<0.001
No	19912	4193 (21.1)		3746 (18.8)	
Yes	9458	1992 (21.1)		1949 (20.6)	
Treatment year			<0.001		<0.001
2003-2009	2527	359 (14.2)		336 (13.3)	
2010-2011	5992	1261 (21.0)		1024 (17.1)	
2012-2013	13379	2832 (21.2)		2700 (20.2)	
2014-2015	7472	1733 (23.2)		1635 (21.9)	
Fertilization method			<0.001		0.224
IVF	20586	4443 (21.6)		3954 (19.2)	
ICSI	8784	1742 (19.8)		1741 (19.8)	
Endometrial preparation for FET			<0.001		<0.001
Natural cycle	8769	1795 (20.5)		1599 (18.2)	
Mild stimulation	10491	1988 (18.9)		1780 (17.0)	
HRT	10110	2402 (23.8)		2316 (22.9)	
Endometrial thickness at transfer (mm)			<0.001		<0.001
≤8	24920	5307 (21.3)		332 (13.4)	
8-15	2476	380 (15.3)		4943 (19.8)	
≥15	1974	498 (25.2)		420 (21.3)	
Embryo stage at transfer			<0.001		0.164
Cleavage stage	25780	5220 (20.2)		4968 (19.3)	
Blastocyst stage	3590	965 (26.9)		727 (20.3)	
No. of embryos transferred			<0.001		<0.001
1	3953	565 (14.3)		470 (11.9)	
2	24136	5417 (22.4)		5035 (20.9)	
3	1281	203 (15.8)		190 (14.8)	

Values are presented in the form number (percentage). BMI, body mass index; PCOS, polycystic ovary syndrome; OPU, ovum pick-up; IVF, in vitro fertilization; ICSI, intracytoplasmic sperm injection; FET, frozen-thawed embryo transfer; HRT, hormone replacement therapy.

Bold indicates statistical significance.

**Table 4:** ORs of live birth of male and female offspring according to patient characteristics and treatment parameters across all transferred cycles.

	Male offspring		Female offspring	
	COR (95% CI)	AOR (95% CI)	COR (95% CI)	AOR (95% CI)
Maternal age				
≤29	Reference	Reference	Reference	Reference
30-34	**0.85 (0.79-0.91)**	**0.89 (0.82-0.96)**	**0.83 (0.78-0.89)**	**0.91 (0.84-0.99)**
35-37	**0.68 (0.62-0.74)**	**0.75 (0.68-0.84)**	**0.64 (0.58-0.70)**	**0.74 (0.67-0.83)**
38-40	**0.41 (0.37-0.47)**	**0.49 (0.42-0.58)**	**0.40 (0.35-0.45)**	**0.50 (0.42-0.58)**
41-43	**0.18 (0.15-0.23)**	**0.23 (0.18-0.29)**	**0.21 (0.17-0.26)**	**0.27 (0.21-0.34)**
≥44	**0.04 (0.02-0.07)**	**0.05 (0.03-0.09)**	**0.03 (0.02-0.06)**	**0.04 (0.02-0.09)**
Maternal BMI				
<18.5	1.06 (0.97-1.16)	0.94 (0.86-1.04)	1.09 (0.99-1.19)	0.98 (0.89-1.07)
18.5-24	Reference	Reference	Reference	Reference
>24	0.96 (0.89-1.03)	1.01 (0.94-1.10)	**0.91 (0.84-0.99)**	0.93 (0.86-1.01)
Male partner age				
≤29	Reference	Reference	Reference	Reference
30-39	**0.86 (0.79-0.92)**	1.02 (0.94-1.12)	**0.79 (0.73-0.85)**	0.97 (0.89-1.07)
40-49	**0.46 (0.41-0.51)**	1.03 (0.90-1.18)	**0.42 (0.38-0.46)**	0.96 (0.84-1.10)
≥50	**0.26 (0.19-0.35)**	0.88 (0.63-1.23)	**0.29 (0.21-0.39)**	0.95 (0.68-1.31)
Duration of infertility (years)				
≤1	Reference	Reference	Reference	Reference
2-4	**0.88 (0.82-0.95)**	**0.86 (0.80-0.94)**	**0.92 (0.85-0.99)**	**0.90 (0.83-0.98)**
5-9	**0.78 (0.71-0.85)**	**0.84 (0.76-0.92)**	**0.78 (0.72-0.86)**	**0.86 (0.78-0.94)**
≥10	**0.46 (0.40-0.53)**	**0.73 (0.63-0.85)**	**0.49 (0.43-0.57)**	**0.81 (0.69-0.94)**
Previous miscarriages				
0	Reference	Reference	Reference	Reference
1	0.94 (0.88-1.01)	1.00 (0.94-1.08)	**0.91 (0.85-0.97)**	0.99 (0.92-1.07)
≥2	**0.78 (0.72-0.94)**	0.94 (0.87-1.03)	**0.74 (0.68-0.80)**	0.92 (0.85-1.01)
Previous ectopic pregnancy				
No	Reference	Reference	Reference	Reference
Yes	**1.16 (1.05-1.28)**	1.00 (0.90-1.12)	**1.17 (1.06-1.30)**	1.05 (0.94-1.17)
PCOS				
No	Reference	Reference	Reference	Reference
Yes	**1.29 (1.15-1.45)**	1.08 (0.95-1.21)	**1.49 (1.33-1.67)**	**1.24 (1.10-1.39)**
Male factor				
No	–	–	Reference	Reference
Yes	**-**	–	**1.12 (1.05-1.19)**	**1.10 (1.07-1.12)**
Treatment year	**1.08 (1.06-1.10)**	**1.08 (1.06-1.10)**	**1.10 (1.09-1.13)**	**1.10 (1.07-1.12)**
Fertilization method				
IVF	Reference	Reference	–	–
ICSI	**0.90 (0.84-0.96)**	**0.90 (0.83-0.97)**	–	–
Endometrial preparation for FET				
Natural cycle	Reference	Reference	Reference	Reference
Mild stimulation	**0.91 (0.85-0.98)**	**0.90 (0.84-0.97)**	**0.92 (0.85-0.99)**	**0.90 (0.83-0.97)**
HRT	**1.21 (1.13-1.30)**	1.05 (0.97-1.13)	**1.33 (1.24-1.43)**	**1.14 (1.06-1.23)**
Endometrial thickness at transfer, mm				
≤8	**0.67 (0.60-0.75)**	**0.78 (0.70-0.88)**	**0.63 (0.56-0.71)**	**0.76 (0.67-0.86)**
8-15	Reference	Reference	Reference	Reference
≥15	**1.25 (1.12-1.39)**	**1.19 (1.06-1.32)**	1.09 (0.98-1.22)	1.02 (0.91-1.15)
Embryo stage at transfer				
Cleavage stage	Reference	Reference	Reference	Reference
Blastocyst stage	**1.45 (1.34-1.57)**	**1.70 (1.56-1.85)**	**1.06 (0.98-1.16)**	**1.26 (1.15-1.39)**
No. of embryos transferred				
1	Reference	Reference	Reference	Reference
2	**1.74 (1.58-1.91)**	**1.95 (1.76-2.15)**	**1.95 (1.77-2.16)**	**1.97 (1.77-2.19)**
3	1.14 (0.96-1.36)	**1.79 (1.47-2.18)**	**1.29 (1.08-1.55)**	**1.95 (1.59-2.38)**

BMI, body mass index; PCOS, polycystic ovary syndrome; OPU, ovum pick-up; IVF, in vitro fertilization; ICSI, intracytoplasmic sperm injection; FET, frozen-thawed embryo transfer; HRT, hormone replacement therapy; COR, crude odds ratio; AOR, adjusted odds ratio; CI, confidence interval. AORs were adjusted for all those covariates with adjusted OR in the table using a binary logistic regression model.

Bold indicates statistical significance.

### Association between risk factors and newborn gender among all live birth of twins

The results of the subgroup analysis are displayed in **Table 5**. When blastocyts were transferred, as opposed to cleavage-stage embryos, the likelihood of delivery of two male twins was significantly higher, whereas the likelihood of delivery of two female twins was noticeably lower (two male twins: aOR 1.78, 95% CI 1.42–2.24; two female twins: aOR 0.64, 95% CI 0.48–0.84).

**Table 5:** AORs for live birth of two male twins and two female twins according to patient characteristics and treatment parameters.

	AOR (95% CI)	
	Two male twins (N=794)	Two female twins (N=683)
Maternal age		
≤29	Reference	Reference
30-34	1.03 (0.83-1.29)	1.03 (0.82-1.29)
35-37	1.11 (0.81-1.53)	0.91 (0.64-1.28)
38-40	1.18 (0.68-2.07)	1.49 (0.84-2.66)
41-43	0.36 (0.08-1.66)	0.96 (0.25-3.65)
≥44	**-**	**-**
Maternal BMI		
<18.5	**0.73 (0.55-0.96)**	**1.48 (1.14-1.92)**
18.5-24	Reference	Reference
>24	0.96 (0.77-1.21)	0.99 (0.78-1.25)
Male partner age		
≤29	Reference	Reference
30-39	0.93 (0.73-1.18)	0.97 (0.75-1.24)
40-49	1.13 (0.75-1.68)	0.70 (0.45-1.10)
≥50	0.58 (0.15-2.20)	2.21 (0.73-6.75)
Duration of infertility (years)		
≤1	Reference	Reference
2-4	0.94 (0.75-1.18)	1.19 (0.93-1.51)
5-9	1.05 (0.80-1.36)	1.31 (0.98-1.73)
≥10	0.81 (0.49-1.35)	1.33 (0.79-2.25)
Previous miscarriages		
0	Reference	Reference
1	1.04 (0.85-1.28)	0.88 (0.71-1.10)
≥2	1.09 (0.84-1.39)	0.91 (0.70-1.19)
Previous ectopic pregnancy		
No	Reference	Reference
Yes	0.88 (0.65-1.20)	1.17 (0.85-1.61)
Tubal factor		
No	Reference	Reference
Yes	1.04 (0.88-1.24)	0.99 (0.83-1.19)
PCOS		
No	Reference	Reference
Yes	1.04 (0.76-1.42)	0.98 (0.71-1.35)
Endometriosis		
No	Reference	Reference
Yes	1.36 (0.98-1.90)	1.05 (0.74-1.51)
Male factor		
No	Reference	Reference
Yes	1.09 (0.89-1.35)	1.04 (0.84-1.30)
OPU Year	0.99 (0.93-1.06)	1.01 (0.95-1.08)
Fertilization method		
IVF	Reference	Reference
ICSI	0.92 (0.73-1.14)	1.04 (0.83-1.31)
Endometrial preparation for FET		
Natural cycle	Reference	Reference
Mild stimulation	1.04 (0.83-1.30)	1.06 (0.84-1.34)
HRT	0.94 (0.77-1.16)	1.08 (0.87-1.34)
Endometrial thickness at transfer, mm		
≤8	0.95 (0.65-1.39)	1.24 (0.85-1.83)
8-15	Reference	Reference
≥15	1.18 (0.88-1.58)	0.91 (0.66-1.26)
Embryo stage at transfer		
Cleavage stage	Reference	Reference
Blastocyst stage	**1.78 (1.42-2.24)**	**0.64 (0.48-0.84)**
No. of embryos transferred		
1	1.09 (0.42-2.85)	**4.32 (1.71-10.93)**
2	Reference	Reference
3	0.96 (0.58-1.59)	0.80 (0.46-1.40)

BMI, body mass index; PCOS, polycystic ovary syndrome; OPU, ovum pick-up; IVF, in vitro fertilization; ICSI, intracytoplasmic sperm injection; FET, frozen-thawed embryo transfer; HRT, hormone replacement therapy; AOR, adjusted odds ratio; CI, confidence interval. AORs were adjusted for all covariates presented in the table using a binary logistic regression model.

Bold indicates statistical significance.

## Discussion

This retrospective cohort study not only confirmed previous findings regarding the association between blastocyst transfer and a skew towards male offspring, as well as the tendency for ICSI to result in fewer male offspring, but also expanded upon these findings by analyzing the rate of live births of each gender. The findings revealed that ICSI was linked to a lower rate of male live births, and that blastocyst transfer favored male live births over female live births.

Jacobsen et al. analyzed a population of over 800,000 babies born in Denmark between 1980 and 1993, establishing a natural reference point for the male-to-female ratio at birth of approximately 51.2% males (5). As summarized in **Table 1**, previous studies have reported male-to-female ratios following IVF ranging from 50.8% males to 52.6% males (18–23). In the present study, the overall male-to-female ratio was 52.3% males, surpassing the figures reported in previous studies. This discrepancy may be attributed to variations in the incidence of various risk factors. Our study involved a lower rate of use of ICSI (29.5%) compared to previous studies (44.8%–61.9%) (18, 19, 22), while a larger proportion of patients in our study underwent blastocyst transfer (14.5%) compared to a study conducted in 2014 (10.9%) (20).

The relationship between maternal age and the male-to-female ratio at birth remains controversial. Rueness et al. have reported a positive association between maternal age and male-to-female ratio at birth, attributing this association to an increased risk of miscarriage related to adverse events during pregnancy in female fetuses (7). Conversely, Matsuo et al. have reported that advanced maternal age is associated with a higher likelihood of female offspring (31). Beyond these two studies, most research has failed to establish a significant relationship between maternal age and the male-to-female ratio at birth (18, 21, 22, 32). In our study, we observed that advanced maternal age was associated with a decreased live birth rate for both genders, but did not influence the final male-to-female ratio.

Our study identifies a possible mechanism underlying the alteration in sex ratio associated with ICSI (19–21, 24). Specifically, we found that ICSI was correlated with a decreased likelihood of male live births, and this may be attributable to selection preference in ICSI procedures. Unlike IVF, ICSI involves the artificial selection of spermatozoa, primarily based on their morphology and motility. A prospective randomized study has shown that intracytoplasmic morphologically selected sperm injection (IMSI), in which a high-magnification microscope is employed for sperm selection, results in a higher proportion of female embryos compared to standard ICSI (66.9% vs. 52.5%, respectively). Additionally, it was observed that morphologically normal spermatozoa were less likely to carry the Y chromosome (33). Consequently, Y-bearing spermatozoa might be less likely to be selected in the artificial selection process involved in ICSI, leading to a reduced chance of male live births. Furthermore, it is noteworthy that oocytes exhibit higher susceptibility to Y-bearing spermatozoa, which suggests that oocytes might have a greater tendency to be fertilized by Y-bearing spermatozoa in IVF compared to ICSI (34–36). However, it is important to note that the advantage of Y-bearing spermatozoa in IVF, in terms of fertilization chance, may be eliminated in ICSI procedures. Although ICSI is commonly recommended for patients with severe male factor infertility, such as severe oligoasthenoteratozoospermia (37), neither previous studies (21, 38) nor our current study have identified any association between male factor infertility and the male-to-female ratio at birth, suggesting that the ICSI procedure itself may act as an independent factor influencing the male-to-female ratio.

In line with previous studies (19, 21, 25–27), our findings also showed that blastocyst transfer was associated with a significantly higher male-to-female ratio at birth compared to cleavage embryo transfer. Remarkably, this association was also observed in the subgroup of all live births of twins. Furthermore, our study revealed a possible previously unrecognized mechanism underlying this association: a sex-related differential response to blastocyst culture *in vitro*. We found that blastocyst culture *in vitro* increased the chance of live birth by 70% for male embryos, whereas the increase for female embryos was only 26%. Two potential explanations for this effect can be considered.

First, it is possible that male embryos exhibit faster growth rates than female embryos, resulting in better morphological grades. Alfarawati et al. found that male embryos were 2.6 times more likely to develop into grade 5 or 6 blastocysts compared to female embryos. Additionally, they reported that among the slowest-growing embryos (grade ≤ 3), 60% (124 of 207) were female, while only 40% (83 of 207) were male (39). A study by Ray additionally showed that male embryos have more cells on Day 2 compared to female embryos (40). Similarly, Pergament et al. observed that the percentage of male embryos with four or more cells on Day 2 was six times higher than that of female embryos (41). Animal studies have indicated that male embryos tend to develop at a faster rate than female embryos, resulting in a higher proportion of good-quality male embryos on Day 3 (42–44). Dumoulin et al. counted blastocyst cell numbers and found that male blastocysts derived from ICSI had more cells than female blastocysts (106.00 ± 9.06 vs. 65.00 ± 9.17, *P* < 0.01) (45). One possible explanation for the delayed development observed in female embryos is their higher requirement for glucose during the pre-implantation stage compared to male embryos (46–48). Although several studies have shown that there is no sex imbalance among blastocysts (39, 49), it is important to consider the selection process of blastocysts for transfer, which was primarily based on morphological criteria, such as cell number and degree of tightness, as well as developmental stage according to the Gardner and Schoolcraft grade system (29). Hence, when embryos are assessed at roughly the same time point, male embryos (with their higher cell count) may tend to receive better grades, potentially resulting in an increased chance of selection of male embryos for transfer.

Second, there is a possibility that the *in vitro* environment may have an adverse effect in terms of X chromosome inactivation (XCI), which in turn may impair the development of female embryos. At the appropriate time, XCI is a crucial step in the normal development of female embryos (50). However, studies have suggested that an unphysiological environment might lead to precocious random XCI in human embryonic stem cells (51). In the context of bovine embryos, Oliveira et al. found that *in vitro* culture was associated with higher expression of XIST, a major controller of XCI, compared to *in vivo* conditions (52). Interference with the appropriate timing of XCI during *in vitro* culture could potentially disrupt the normal process of implantation and development, and even lead to early embryonic death.

To the best of our knowledge, this study is the first to explore the influence of risk factors on the live birth rate for each gender, providing new insights into the mechanisms underlying the skewed male-to-female ratio associated with IVF/ICSI and FET. Another key strength of this study lies in the comprehensive exploration of the association between the male-to-female ratio at birth and various factors involved in ART, including the clinical characteristics of infertile couples and treatment interventions, on which is there is little information in the existing literature. Additionally, the relatively large sample size of this study ensures more reliable modeling and reduces potential bias.

The major weakness of this study is its retrospective and non-randomized design, which introduces the possibility of unknown confounding factors. In addition, data on known confounders such as adverse environmental exposure and psychological conditions (14, 16, 17) were not available in our database. Another limitation is the absence of data on the gender of embryos with an outcome of embryonic death or miscarriage, which constrains further exploration of the underlying mechanisms contributing to the gender bias.

## Conclusion

ICSI was found to be associated with a decreased male-to-female ratio and a lower rate of male live births in FET cycles, while blastocyst transfer was associated with an increased male-to-female ratio at birth and a higher likelihood of male live birth compared to female live birth.

## Data availability statement

The data underlying this article cannot be shared publicly to protect the privacy of individuals included in the study.

## Author contributions

TD, BL, and ZY conceived and designed this study. TD, JQ, SZ, JL, and DZ participated in data collection. TD, QX, JY, SZ, JL, and DZ analyzed and interpreted the data. QX, TD, XW, JL, and DZ participated in drafting the manuscript. All the authors participated in making revisions to the article, as well as reading and approving the final manuscript.
